# Decomposition of triazole and 3-nitrotriazole upon low-energy electron attachment†

**DOI:** 10.1039/d3cp01162c

**Published:** 2023-04-27

**Authors:** Muhammad Saqib, Farhad Izadi, Leon U. Isierhienrhien, Milan Ončák, Stephan Denifl

**Affiliations:** a Institut für Ionenphysik und Angewandte Physik, Universität Innsbruck Technikerstraße 25 A-6020 Innsbruck Austria milan.oncak@uibk.ac.at stephan.denifl@uibk.ac.at; b Center for Biomolecular Sciences Innsbruck, Universität Innsbruck Technikerstraße 25 A-6020 Innsbruck Austria

## Abstract

Compounds based on nitrotriazole have been studied for their application as potential radiosensitizers for the treatment of tumors and as energetic materials. In the former application, the initial reduction of the compounds may serve as a mechanism which leads to the formation of tumor-active species. In this study, we investigated the fundamental properties of anion formation in isolated 3-nitro-1,2,4-triazole (3NTR) molecules upon attachment of low-energy electrons. The resulting product anions formed were detected *via* mass spectrometry. Quantum chemical calculations were performed to study the dissociation pathways and to derive the threshold energies. We also studied the attachment of electrons to the native 1*H*-1,2,4-triazole (TR) molecule, revealing the influence of the nitro group on anion formation. Comparing the results for these two systems, we computationally observed a considerable more stable parent anion for 3NTR, which results in significantly more effective degradation of the molecule at lower electron energies. Although characteristic fragmentation reactions in the presence of the nitro group were observed (like formation of NO_2_^−^ or the release of an OH radical), the main dissociation channel for the 3NTR anion turned out to be the direct dissociation of a hydrogen radical by a single bond cleavage, which we also observed for TR as the main channel. Thus, the triazole ring shows a pronounced stability against electron attachment-induced cleavage compared, for example, to the imidazole ring, which is found in common nitroimidazolic radiosensitizers.

## Abstract 2

The addition of the NO_2_ group to the triazole ring promotes the efficiency of electron attachment. Low-energy electrons decompose the molecule at sub-excitation energies.

## Graphical abstract

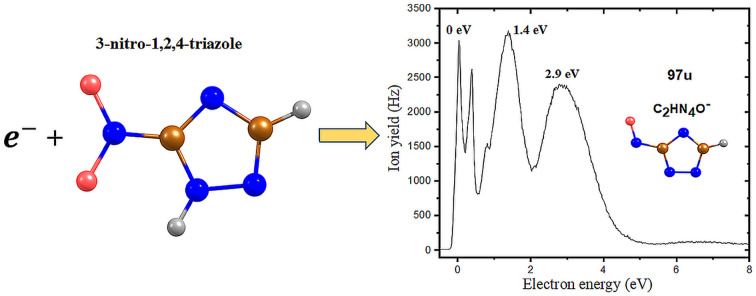

## Introduction

Triazole (C_2_H_3_N_3_) belongs to the group of heterocyclic azoles and contains three nitrogen atoms in a five-membered ring.^1^ With its two possible isomeric configurations, 1,2,3-triazole and 1,2,4-triazole derivatives of this compound find a very broad spectrum of applications in medical treatment,^2–4^ for example, as antifungal,^5^ antiflammatory,^6^ anti-oxidative^7^ and anticancer drugs.^8^ Triazoles also play a fundamental role in chemistry since 1,2,3-triazole is a product of Huisgen azide–alkyne cycloaddition with copper as a catalyst.^9^ The latter reaction represents a prime process of click chemistry, which aims to form substances by covalently joining smaller entities together.^10^ For example, triazole-linked analogues of DNA were synthesized, which pointed out new pathways of oligonucleotide chemistry by replacing the native phosphate-linking moiety.^11,12^ Another study pointed out that triazoles are excellent π-conjugative bridges for (photoinduced) electron transfer between electron donors and acceptor moieties.^13^ Therefore, the electronic properties of triazoles attracted particular attention.^14–16^ The favorable characteristic towards electron transport in materials also suggests the functionalization of various nanostructures and nanoparticles for different applications like for example CO_2_ capture^17^ and optoelectronics.^18,19^ Therefore, it was pointed out that triazole has been considered as one of the most important functional heterocyclic systems in modern chemistry.^4^

The replacement of hydrogen at the C3 position by a nitrogen dioxide (NO_2_) group represents one of the highly potential modifications of the triazole molecule. In addition to their use in medical and functional applications, nitrotriazole-based compounds were also employed as energetic materials.^20^ Explosives like trinitrotoluene carry the hazard of inadvertent detonations and therefore attempts to replace standard compounds are undertaken. Meanwhile, nitrotrizolone (3-nitro-1,2,4-triazol-5-one, C_2_H_2_N_4_O_3_) is considered as a main component for less sensitive explosive formulations.^21^ Since the knowledge of its toxicity is limited, concerns on its implications on environmental physics/chemistry are raised and hence there is necessity for its detection methods and development of degradation pathways. Studies on possible UV photodegradation of nitrotrizolone in wastewater (also with the “chemical support” by hydrogen peroxide)^22,23^ as well as molecular degradation in solid bioreactors were carried out previously.^21^ In the latter case, the favorable electronic properties of the nitro-compound may be exploited for initial microbial reduction under anaerobic conditions which can also be achieved in an otherwise bulk aerobic soil environment. Interestingly, under particular substrate conditions, the formation of new high-nitrogen azo and azoxy compounds upon the electrochemical reduction of nitrotriazoles in aqueous media was reported as well.^24^ This result suggested that electrochemical reduction may be an economic way for the treatment of waste containing nitrotriazole-based compounds by forming new green energetic materials rich of nitrogen.^24^

Another area in which favorable reduction properties of nitrotriazole could be very useful is related to radiosensitization of tumor cells. An early cell culture study suggested that 3-nitrotriazole (C_2_H_2_N_4_O_2_) has potential as a hypoxic cell sensitizer.^25^ Interestingly, the sensitizing efficiency of nitrotriazole was estimated to be higher by a factor of 100 than that of nitroimidazole in which the electron-withdrawing NO_2_ group is attached to an imidazole ring. From the chemical perspective, this effect of higher sensitivity of the triazole compound was explained by the additional presence of a pyridine type nitrogen atom, which leads to a decrease of the π-density of the heterocyclic system and a subsequent increase of the electron affinity.^25^ Further *in vitro* and *in vivo* studies with nitrotriazole compounds^26,27^ supported the initial conclusion of a radiosensitizing effect in cells, paired with a relatively low toxicity.^28,29^ Activation of these compounds in cells may happen by initial reduction,^30^ either from enzymes or by attachment of low-energy electrons.^31^ The latter are formed in abundant amounts upon the interaction of high-energy radiation with biological matter and their initial energy distribution peaks around 9–10 eV.^32^ The kinetic energy of these low-energy electrons becomes reduced in a series of elastic and inelastic collisions within the medium before they reach thermal energies.

In the present study, we investigated electron attachment to the 1*H*-1,2,4-triazole (TR) molecule and 3-nitro-1,2,4-triazole (3NTR) in the gas phase using mass spectrometric methods and quantum chemical calculations. In contrast to other basic heterocyclic aromatic compounds of biological relevance, like furan,^33^ imidazole,^34^ pyridine,^35^ purine,^36^ and pyrimidine,^37^ to the best of our knowledge, no comprehensive study of anion formation and possible dissociation pathways exists so far for triazole in the gas phase. The same situation applies to nitro-triazoles, while electron attachment data for the nitro-analogues nitroimidazole,^38^ 2-nitrofuran^39^ and 5-nitrouracil^40^ are available in the literature. Our results show how the presence of the NO_2_ group at the triazole ring changes the dissociation pathways considerably, increases the electron affinity and opens a multitude of new dissociation channels which are mostly connected to processes directly involving NO_2_ group predissociation.

## Experimental and computational methods

The electron attachment study in the gas phase was carried out using a crossed electron-molecular beam instrument.^31^ The basic components of this setup are (i) the neutral beam source, (ii) an electron source, (iii) a quadrupole mass spectrometer, and (iv) the ion detection system. Before starting the measurements, two different inlets have been tested to achieve an effusive molecular beam under high vacuum conditions (∼10^−8^ mbar background pressure). One possibility includes an oven with a glass inset placed in the main vacuum chamber. The vapor of the sample sublimated in the oven is guided with a copper capillary of 1 mm inner diameter to the interaction with the electron beam. In the current experiments, the 3NTR sample (purchased from Merck, Vienna, Austria, with the stated purity of 97%) was placed in the oven. For the negative ion measurements, the sample was heated up to about 366 K.

For the TR sample (also purchased from Merck, Vienna, Austria, with the stated purity of 98%), an external sample container was used instead. In this case, the sample container is located outside the vacuum chamber and is connected to an inlet valve system in order to control the gas amount introduced in a 1 mm diameter capillary tube (made of stainless steel) to the interaction region of the electron beam. The TR sample was heated to about 329 K for the negative ion measurements. For both samples, the measurement of the electron ionization mass spectrum at 70 eV was used to rule out thermal decomposition as well as impurities affecting the reported negative ion yields. In these positive ion studies, the temperature dependence of the mass spectrum was followed in the temperature range of 318–357 K and 263–370 K for TR and 3NTR, respectively. The working pressure in the vacuum chamber was 2.8 × 10^−7^ mbar for TR and 1.2 × 10^−7^ mbar for 3NTR.

The formed effusive beam entered the interaction region of the hemispherical electron monochromator (HEM). The HEM comprises three basic elements, (i) a hairpin tungsten filament which is heated by applying a current of about 2.35 A and generates the electron beam, (ii) two concentric hemispheres at different electric potentials, which lead to an electric field used for the selection of the kinetic energy of the electrons, and (iii) two stacks of electrostatic lenses. The latter component is used for the guidance of the electron beam from the filament to hemisphere and from the hemisphere to the interaction region. In the course of the present experiments, an electron beam, with about 70–100 nA current, was directed to the interaction region with the neutral molecules. The energy resolution of the electron beam was about 130 meV for full width at half maximum (FWHM). This FWHM was determined by measuring the well-known sharp 0 eV resonance of Cl^−^ formed upon electron attachment to CCl_4_ under the same experimental conditions.^41^ The amount of electron current was collected with a Faraday cup right after the interaction region. The cup was connected with a picoampere meter. The anions created in the interaction region were extracted by a weak electric field and directed towards a quadrupole mass spectrometer with a nominal mass range of 2048 u. After the quadrupole, the mass-analyzed ions were deflected by 90° with an electrostatic deflector and detected by a channeltron. For recording the anion efficiency curves shown in this work, the corresponding mass of the anion was set using the quadrupole and the electron energy was repeatedly scanned in a chosen electron energy range. The onset of the measured ion yield was derived by the method introduced in ref. 42.

To support the experiment, quantum chemical calculations were performed. Optimization of structures was performed employing density functional theory (DFT) using the B3LYP functional along with the aug-cc-pVTZ basis set, and the nature of local minima and transition states was confirmed through frequency analysis. To obtain more reliable energetics, a single-point coupled cluster calculation with singles, doubles, and non-iteratively included triples, CCSD(T), with the same basis set was performed for the obtained structures. The zero-point correction was included at the B3LYP/aug-cc-pVTZ level. To describe the dipole-bound state in TR, the aug-cc-pVTZ basis set was augmented for hydrogen atoms by two s functions and one p and one d function, with coefficients determined as one third of the lowest coefficient in the basis set, denoted as aug-cc-pVTZ(H+). Population analysis was performed using the charges from electrostatic potentials using a grid-based method (CHELPG) scheme.^43^ To analyze the electronically excited states in anions, we employed time-dependent DFT (TDDFT) with the CAM-B3LYP functional, complete active space self-consistent field (CASSCF), and multi-reference configuration interaction (MRCI). We picked the active space of 3 electrons in 5 orbitals, further denoted as (3,5). To describe the three lowest electronic states in 3NTR^−^, the singly occupied orbital and two unoccupied orbitals would be sufficient, and two more orbitals were added for flexibility. The average over three electronic states was employed. The aug-cc-pVDZ basis set was de-contracted for multi-reference calculations to allow for optimization of the conical intersection. Wave function stability was checked prior to each calculation. CASSCF and MRCI calculations were performed in Molpro,^44,45^ and the other calculations were performed using the Gaussian program.^46^

## Results and discussion

**Fig. 1:**
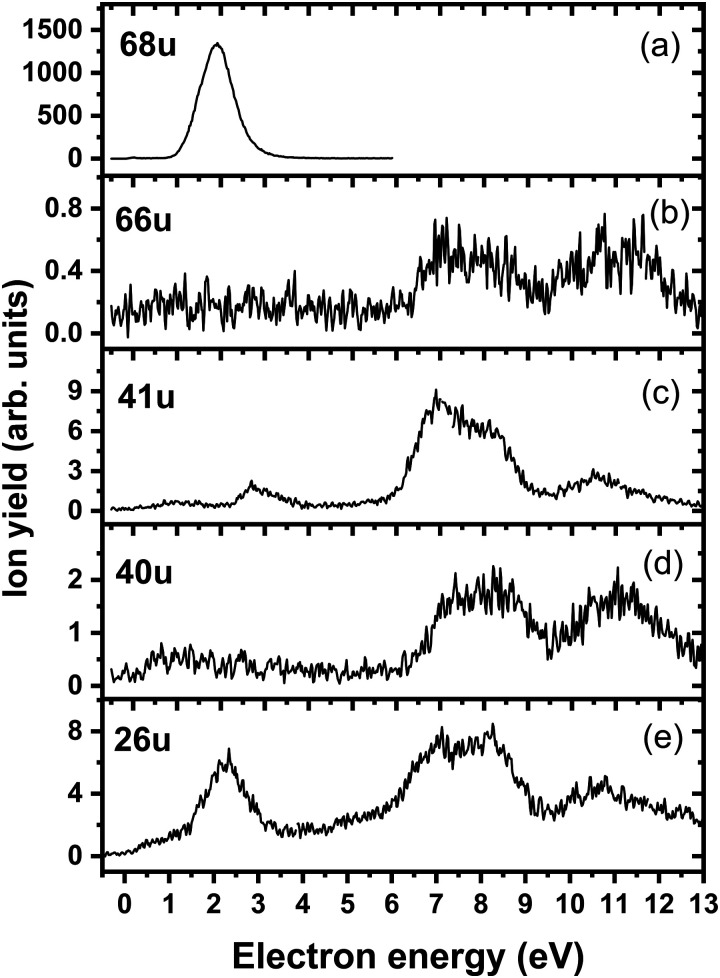
Anion efficiency curves of the anions with masses of (a) 68 u, (b) 66 u, (c) 41 u, (d) 40 u, and (e) 26 u, formed upon electron attachment to triazole.

**Fig. 2:**
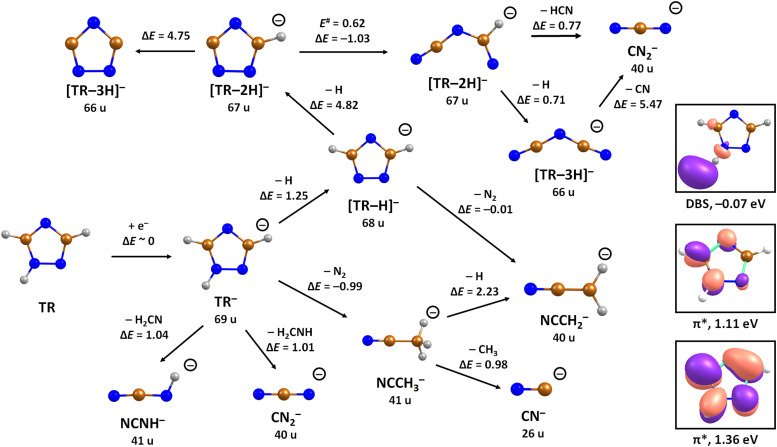
Suggested dissociation pathways in triazole along with reaction energies. Calculated at the CCSD(T)/aug-cc-pVTZ//B3LYP/aug-cc-pVTZ level of theory. The orbitals occupied by the attached electron in the dipole-bound state (DBS), and two lowest-lying valence states of TR^−^ are shown as calculated at the (TD-CAM-)B3LYP/aug-cc-pVTZ(H+) level. Colour code: blue-nitrogen, brown-carbon, grey-hydrogen.

We start our discussion with the analysis of DEA to triazole, TR. The anion efficiency curves of the fragment anions detected are shown in Fig. 1. Table 1 summarizes the corresponding anion assignments and the peak positions as well as the experimental threshold derived from the data. The molecule is calculated to have very low vertical and adiabatic electron affinities of 0.07 eV and 0.08 eV at the B3LYP/aug-cc-pVTZ(H+) level (the values are close to 0 eV upon single-point CCSD(T) recalculation). The lowest-lying electronic state upon electron attachment has a dipole-bound character, with the attached electron located close to the N–H bond, as depicted in Fig. 2 (dipole moment of the TR molecule is calculated as 2.8 Debye, making the formation of a dipole-bound state possible). Due to this low binding energy of the excess electron, detection of such parent anion on typical mass spectrometric timescales (requires lifetimes > μs) is unlikely under single collision conditions. Instead, fast spontaneous emission of the excess electron or coupling of the dipole bound state with a valence bound state may occur.^47^ Such coupling may also act a doorway mechanism to dissociation, as discussed for biomolecules previously.^48,49^ The lowest electronic states of valence character are predicted to be two π* states, see Fig. 2 (TD-CAM-B3LYP/aug-cc-pVTZ(H+) level).

**Table 1:** Summary of the peak positions and experimental thresholds *E*_trh_, and calculated reaction energies *E*_calc_ corresponding to dissociation reactions from TR + e^−^ producing an anion and one or several neutral molecules as shown in Fig. 2 (all in eV) for the fragment anions formed upon electron attachment to TR. Reaction energies were obtained at the CCSD(T)/aug-cc-pVTZ//B3LYP/aug-cc-pVTZ level

Mass (u)	Anion	Peak positions						*E*_thr_	*E*_calc_
		1	2	3	4	5	6		
68	C_2_H_2_N_3_^−^	1.9	—	—	—	—	—	1.1	1.25
66	C_2_N_3_^−^	7.1	8.2	11.0	—	—	—	∼6.1	5.75
41	CHN_2_^−^/C_2_H_3_N^−^	1.1	2.8	6.9	8.1	10.6	—	∼0.4	1.04/−0.99
40	CN_2_^−^/C_2_H_2_N^−^	7.2	8.4	11.1	—	—	—	∼6.2	1.01/1.24
26	CN^−^	0.9	2.3	5.3	6.9	8.2	10.8	∼0	–0.01

Upon DEA to triazole, five fragment anions are observed in the experiment (Fig. 1). The (dipole-bound) parent anion, TR^−^, was not recorded as expected. In general, there are three ranges in which the TR molecule attaches electrons, namely 1–3 eV, 6–9 eV, and 10–13 eV. The absence of any low-lying sharp features indicating a vibrational progression may also lead to the conclusion that no doorway mechanism including the dipole-bound anion is operative. Thus, we suggest that only shape resonances are involved in the lowest energy range with the possible electronic excitation of the neutral upon electron attachment at higher electron energies (core-excited resonances). For comparison, the reported lowest electronic excitation energies for 1,2,3-triazole and 1-methyl-1,2,4-triazole are 5.37 eV^50^ and 5.94 eV,^51^ respectively. These values would be near to the onset of the intermediate energy range of resonances observed. We just note that the excitation to Rydberg states may be possible above ∼7 eV as well.^51^ A detailed suggestion for the assignment of core-excited resonances of TR was recently made by Tseplin *et al.*^52^

The most intense fragment anion detected in the present experiment has a mass of 68 u and represents the dehydrogenated triazole parent anion, [TR–H]^−^, formed in a single peak at ∼1.9 eV and no attachment at higher electron energies (see Fig. 1a). All other fragment anions have at least 100× lower intensity. The fragment anion with mass 66 u, assigned to [TR–3H]^−^, shows low yields above 6 eV (Fig. 1b). The fragment anion with mass 41 u, which could be assigned as either CHN_2_^−^ or C_2_H_3_N^−^, shows at least 5 peaks (Fig. 1c), covering the whole ∼0.4–13 eV range. The fragment anion with mass 40 u, either CN_2_^−^ or C_2_H_2_N^−^, has similar features as observed for 66 u, suggesting that these anions might have a common precursor. The weak intensity observed below the electron energy of about 6 eV in Fig. 1d, showing a rather non-resonant behavior, may be assigned to the background signal. We ascribe this intensity to be formed from scattered electrons in the interaction region with the neutral beam. These scattered electrons may be extracted into the direction of the quadrupole mass spectrometer and achieve the required kinetic energy to form CN_2_^−^ or C_2_H_2_N^−^. Finally, the fragment anion with mass 26 u, interpreted as CN^−^, is shown in Fig. 1e and has many similarities in its spectrum with the anion having mass 41 u. Tseplin *et al.* also derived the anion efficiency curves from the negative ion mass spectra from TR using a magnetic mass spectrometer.^52^ Without going into details, the agreement to the present results is reasonable, though a few more weakly abundant fragment anions (like [TR–2H]^−^) and partially other intensity ratios (like for CN^−^) are reported in ref. 52.

To analyze dissociation pathways upon electron attachment to TR, we performed quantum chemical calculations. The results are shown in Fig. 2. The calculated reaction energies are generally in good agreement with the thresholds observed in the experiment, as shown in Table 1.

The dehydrogenated triazole molecule, [TR–H]^−^ or C_2_H_2_N_3_^−^, can be produced starting at ∼1.25 eV by direct dissociation of the N–H bond as shown in Fig. 2. It is difficult to pinpoint the particular reaction pathway as one has to employ a large basis set to describe the electronic ground state of the anion, leading to a number of low-lying pseudo-continuum states. Our simplified calculations at the (TD-CAM-)B3LYP level shown in Fig. S1 (ESI†) show that in the ground state, the single electron is localized in the σ*(N–H) orbital already at the N–H distance of about 1.4 Å. A conical intersection with the electronic state populated upon electron attachment must be reachable already for electron energies of ∼1 eV, as obtained from the anion efficiency curve shown in Fig. 1a. However, in the following discussion, we assume that dissociation takes place from the ground electronic state for low-lying resonances. Description of reaction pathways resulting from shape and core-excited resonances is beyond the scope of the current work.

If the electron brings more energy, further dissociation might take place. First, the second hydrogen atom leaves the molecule requiring about 4.8 eV, possibly leading to the opening of the cycle over a small barrier (upper part of Fig. 2). The resulting anion with mass 67 u is not observed in the experiment, most probably because it dissociates a third H atom to produce a very stable dicyanamide anion, NCNCN^−^, with 66 u. This anion is observed in the experiment at higher energies and is calculated to be by 5.1 eV more stable compared to the structure in which the cycle is kept, *i.e.* the cyclic structure of [TR–3H]^−^. In the latter case, the expected threshold would be 10.8 eV, which is only near the second peak maximum of [TR–3H]^−^. Thus, we rather exclude the formation of the cyclic anion also at such high electron energies. Ring opening reactions were also suggested in multiple dehydrogenation reactions upon attachment of electrons to the imidazole molecule.^34^ However, for imidazole, the fragment anions associated with the subsequent dissociation of ring were about a factor 3 times more intense than dehydrogenation reactions. This shows the higher stability of the triazole ring compared to imidazole. Another possibility for the formation of the [TR–3H]^−^ ion would be dissociation of H_2_ + H, shifting the reaction energy down by the H_2_ binding energy (4.48 eV);^53^ however, H_2_ formation seems less probable due to kinetic effects and the absence of the [TR–2H]^−^ ion in the experiment.

At low electron energies, the formation of NCCH_3_^−^ from TR^−^ through hydrogen transfer and dissociation of N_2_ is possible within an exothermic channel with −0.99 eV, producing an experimentally observed anionic fragment with mass 41 u. From this structure, either H or CH_3_ loss leads to the formation of anions NCCH_2_^−^ (40 u) or CN^−^ (26 u), respectively. The energetically low-lying dissociation of CH_3_ to form CN^−^ might explain the relatively high abundance of CN^−^ and the absence of NCCH_3_^−^ ions in the 1–3 eV region. The NCCH_2_^−^ anion might be also formed through the direct dissociation of N_2_ from [TR–H]^−^ that is practically thermoneutral but requires considerable cluster rearrangement. At higher electron energies, NCNH^−^ and NCN^−^ anions with mass 41 u and 40 u, respectively, might be formed through other dissociation reactions, with CN^−^ possibly formed in further dissociation steps.

**Fig. 3:**
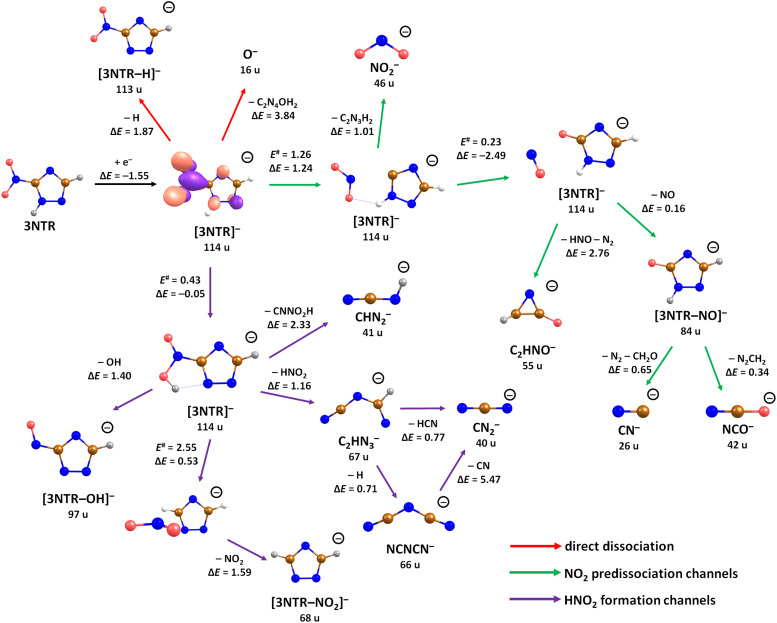
Suggested dissociation pathways in 3-nitrotriazole along with reaction energies. Calculated at the CCSD(T)/aug-cc-pVTZ//B3LYP/aug-cc-pVTZ level of theory. For 3NTR^−^, the orbital occupied by the attached electron is shown as calculated at the B3LYP/aug-cc-pVTZ level. For the transfer of oxygen from NO_2_ to the carbon atom of the triazole ring, NO_2_ reorientation during its roaming is ignored and the transition state for the oxygen atom transfer is used (*E*^#^ = 0.23 eV). Color code: blue-nitrogen, brown-carbon, grey-hydrogen, red-oxygen.

Replacing the hydrogen atom at C3 position in TR by a nitro group to form 3-nitrotriazole, 3NTR, significantly changes both electron attachment properties and dissociation pathways. First of all, the presence of the nitro group increases the electron affinity considerably, with vertical and adiabatic electron affinity of 3NTR calculated as 1.01 and 1.55 eV, respectively. In the ground state of the resulting anion, the electron is localized mainly in the π*(NO_2_) orbital as shown in Fig. 3.

**Fig. 4:**
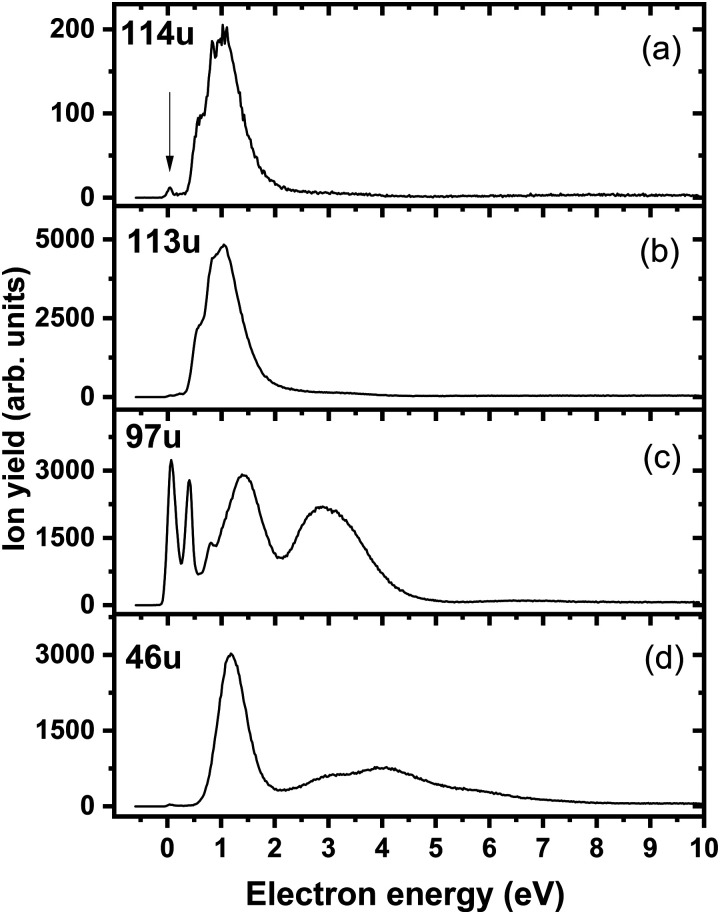
Anion efficiency curves of the anions with masses of (a) 114 u, (b) 113 u, (c) 97 u, and (d) 46 u, formed upon electron attachment to 3NTR. The vertical arrow in panel (a) points to the zero peak, which we assign to the formation of the parent anion of 3NTR. The other signals found for this mass can be ascribed to the isotope of the dehydrogenated parent anion.

**Fig. 5:**
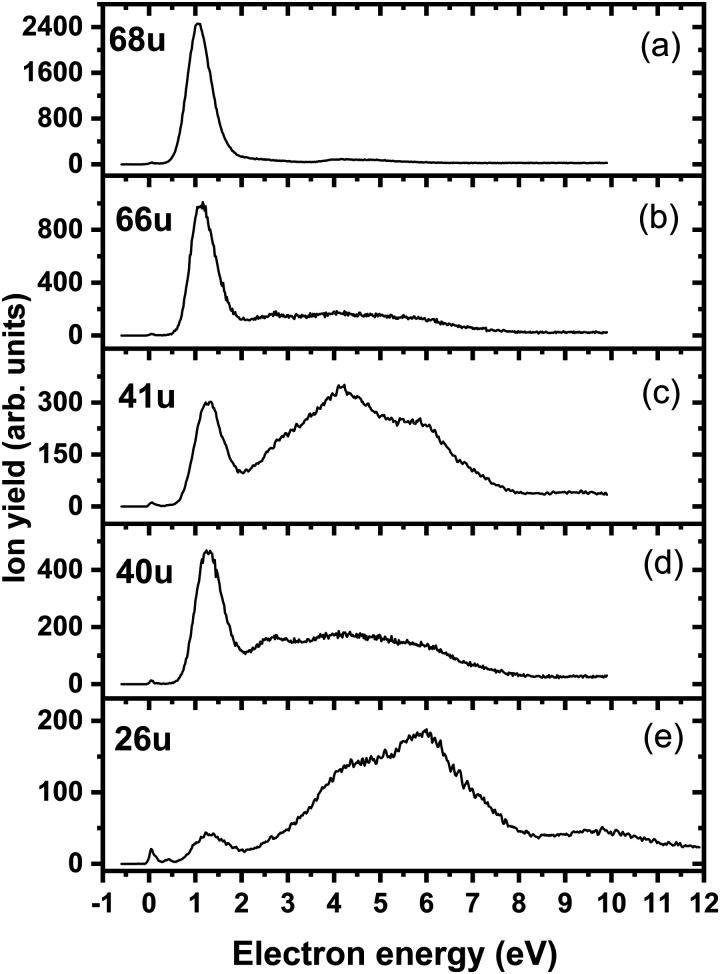
Anion efficiency curves of the anions with masses of (a) 68 u, (b) 66 u, (c) 41 u, (d) 40 u, and (e) 26 u, formed upon electron attachment to 3NTR. Fragment anions with the same masses are also observable for native TR, as shown in Fig. 1 and text.

**Fig. 6:**
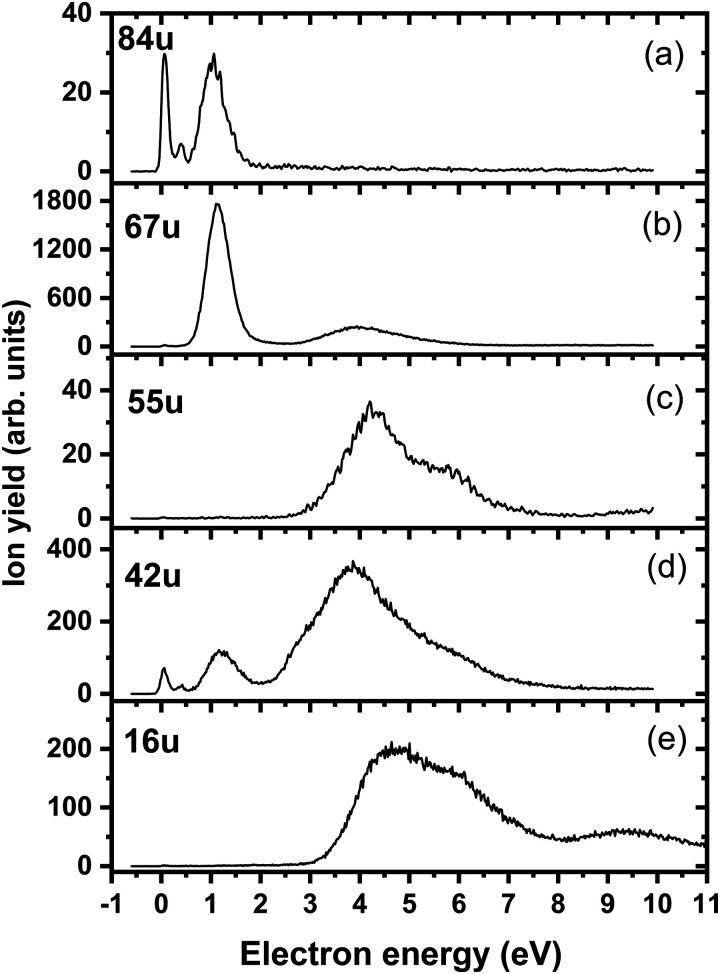
Anion efficiency curves of the anions with masses of (a) 84 u, (b) 67 u, (c) 55 u, (d) 42 u, and (e) 16 u, formed upon electron attachment to 3NTR.

Fig. 4a shows the ion yield measured at the mass of parent anion (114 u). We assign only the weakly abundant zero eV peak to the parent anion, while the signal at higher electron energies can be ascribed to the isotope signal of [3NTR–H]^−^, which is the most abundant anion observed in DEA to 3NTR. The ion yield of [3NTR–H]^−^ with mass 113 u is shown in Fig. 4 (panel (b)), together with the anions efficiency curve for the anions with masses 97 u (Fig. 4c) and 46 u (Fig. 4d). These three fragment anions are the most abundant ones observed in the present experiment. In total, we observed 13 anionic fragments in DEA to 3NTR. Fig. 5 shows the anion efficiency curves of fragment anions with the same nominal mass like those observed for TR. Except for the anion with mass 68 u (C_2_H_2_N_3_^−^), the anion yields are substantially altered upon the addition of the NO_2_ group (panels b–e). Fig. 6 finally shows the anion yields of other anions observed in DEA to 3NTR, which are lower in intensity and were not formed in DEA to TR as well. Table 2 summarizes the peak positions of anions formed in DEA to 3NTR. With the exception of the fragment anions with masses at 55 u and 16 u, all formed fragment anions show a peak near 1 eV, which indicates a common precursor resonance. For four fragment anions (masses 97 u, 84 u, 42 u and 26 u), a structure with a narrow peak at ∼0 eV and ∼400 meV is observed, which may point towards a possible presence of a dipole-bound state (see discussion above); the dipole moment of 3NTR is calculated as 2.8 Debye. Some other fragment anions reveal a very weakly abundant (single) peak at zero eV as well, as shown in Fig. 4–6, which may be rather ascribed to hot band transitions.^54^

**Table 2:** Summary of the peak positions and experimental thresholds *E*_trh_, and calculated reaction energies *E*_calc_ corresponding to dissociation reactions from 3NTR + e^−^ producing an anion and one or several neutral molecules as shown in Fig. 3 (all in eV) for the fragment anions formed upon the attachment of electrons to 3NTR. Reaction energies were obtained at the CCSD(T)/aug-cc-pVTZ//B3LYP/aug-cc-pVTZ level

Mass (u)	Anion	Peak maxima							*E*_thr_	*E*_calc_
		1.	2	3	4	5	6	7		
114	3NTR^−^	0	—	—	—	—	—	—	0	−1.55
113	[3NTR-H]^−^	0.5	0.8	1	2.6	—	—	—	0.4	0.31
97	[3NTR-OH]^−^	0	0.4	0.8	1.4	2.9	—	—	0	−0.20
84	[3NTR-NO]^−^	0	0.4	1	1.25	—	—	—	0	−2.65
68	[3NTR-NO_2_]^−^	1.1	4.1	4.8	—	—	—	—	0.5	0.52
67	C_2_HN_3_^−^	1.1	4.0	—	—	—	—	—	0.6	−0.44
66	C_2_N_3_^−^	1.1	2.7	4.0	5.1	—	—	—	0.6	0.27
55	C_2_HNO^−^	4.3	5.7	—	—	—	—	—	3.0	−0.05
46	NO_2_^−^	1.1	2.7	4.0	5.9	—	—	—	0.5	0.70
42	NCO^−^	0	0.4	1.2	3.8	5.5	—	—	0	−2.31
41	CHN_2_^−^	1.3	3.2	4.2	5.9	9.4	—	—	0.6	0.73
40	CN_2_^−^	1.2	2.5	3.8	5.5	—	—	—	0.6	0.33
26	CN^−^	0	0.4	1.3	4.5	6.0	7.1	9.6	0	−2.00
16	O^−^	4.7	5.6	9.4	—	—	—	—	3.3	2.29

**Fig. 7:**
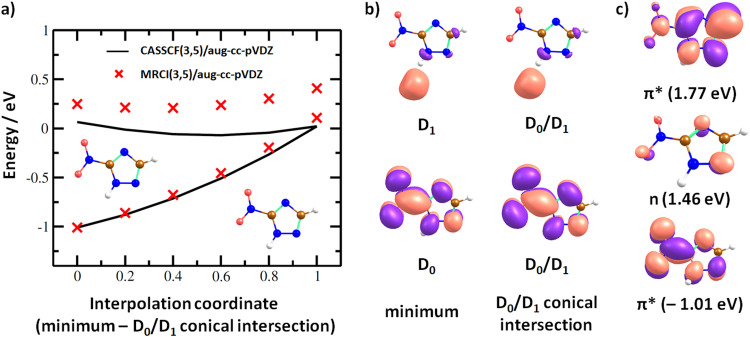
(a) Interpolation in 3NTR^−^ along a coordinate connecting the minimum of the neutral molecule calculated at the B3LYP/aug-cc-pVTZ level and D_0_/D_1_ conical intersection found at the CASSCF(3,5)/aug-cc-pVDZ level of theory. Energy is given with respect to the neutral 3NTR molecule, employing the vertical electron affinity calculated at the CCSD(T)/aug-cc-pVTZ//B3LYP/aug-cc-pVTZ level. (b) Orbitals occupied with a single electron in 3NTR^−^ as calculated at the CASSCF(3,5)/aug-cc-pVDZ level for the minimum of the neutral molecule and the D_0_/D_1_ conical intersection used for the interpolation. (c) Orbitals occupied with a single electron in 3NTR^−^ for selected electronic states and energies with respect to the neutral molecule as calculated at the (TD-)CAM-B3LYP/aug-cc-pVTZ(H+)//B3LYP/aug-cc-pVTZ level of theory.

In the dipole-bound state formed at ∼0 eV, the electron is again localized next to the N–H bond. Fig. 7a shows that upon forming the dipole-bound state, the electron might transfer to the π*(NO_2_) orbital through a low-lying D_0_/D_1_ conical intersection that is directly accessible from the optimal structure of the neutral molecule. The conical intersection has a very similar structure as the minimum of the neutral molecule, with the main difference being prolongation of the C–NO_2_ bond and shortening of the N–O bonds, destabilizing the ground electronic state of the π*(NO_2_) character. Furthermore, two states of valence character in *A*′′ irreducible representation are found at ∼1.5–1.8 eV with respect to the neutral molecule, as shown in Fig. 7c. In the following section, we expect that the ground electronic state of the anion is eventually reached for energetically low-lying resonances, and dissociation proceeds from this state.

The calculated dissociation pathways of 3NTR^−^ are shown in Fig. 3. As the additional electron is located in the π*(NO_2_) orbital in the ground state of the anion, the N–O bond lengths increase from 1.21 Å and 1.23 Å to 1.28 Å and 1.30 Å, respectively. Due to the high electron affinity, the parent anion might stabilize, although its intensity is very low compared to the fragment anions. Note that a (significant) peak near 0 eV is observed only for the ions that are calculated to be formed within exothermic dissociation channels; this result indicates very good agreement of experimental data and computations. In the following section, we distinguish three dissociation pathways, starting with either NO_2_ predissociation/roaming or HNO_2_ formation and direct dissociation channels.

### Predissociation of NO_2_

Upon electron attachment, the NO_2_ group might predissociate over a barrier below the energy of the neutral 3NTR molecule (pathways marked with green arrows in Fig. 3). The pre-dissociated NO_2_ moiety bears the charge of −0.61 *e* and might subsequently form NO_2_^−^ at 46 u, a very intense fragment observed in the experiment.

If the NO_2_ moiety does not dissociate directly, it may roam in the vicinity of the C_2_H_2_N_3_ fragment, in a similar manner as suggested for the other moieties mentioned previously for tirapazamine^55,56^ or 5-fluorouracil.^57^ Various rearrangement options are available for the predissociated NO_2_ moiety (not shown in Fig. 3). Eventually, an oxygen atom might be transferred to a nitrogen atom of the cycle, forming a NO molecule and the [3NTR–NO]^−^ anion with mass 84 u, which has an overall reaction energy of −2.65 eV. Despite the exothermicity of its formation, the intensity of this anion is lower than 1% of the most intense signal. This can be explained by the roaming of NO_2_ needed to form the fragment.

From the [3NTR–NO]^−^ anion, NCO^−^ with mass 42 u or CN^−^ with mass 26 u could also be formed in exothermic reactions through further dissociation of N_2_ and CH_2_O or CH_2_N_2_, respectively. Also, the dissociating NO group might attach an H atom from the cycle, forming HNO, with further N_2_ dissociation disrupting the cycle. This leads to C_2_HNO^−^ with mass 55 u, with an overall reaction energy of −0.05 eV. The anion is observed at higher energies in the experiment most probably due to the rearrangement needed to reach the final anionic structure.

### Formation of HNO_2_

After electron attachment, hydrogen transfer from the N–H group to the NO_2_ group may take place (pathways shown in violet arrows in Fig. 3). This reaction occurs over a barrier of 0.43 eV with respect to the [3NTR]^−^ anion (below the energy of the neutral molecule), *i.e.* the hydrogen atom might move freely between both atoms after the electron is attached. From the HONO group, OH might dissociate directly, with the overall energy of −0.20 eV with respect to the neutral molecule, explaining the high intensity of the [3NTR–OH]^−^ anion with mass 97 u in the experiment.

The HONO group might transfer the hydrogen atom to the carbon atom of the triazole cycle, dissociating as a neutral NO_2_ moiety and producing the [3NTR–NO_2_]^−^ anion observed in the experiment (mass 68 u). The respective transition state lies about 0.4 eV above the experimental threshold of the [3NTR–NO_2_]^−^ anion; the hydrogen transfer might however also take place upon HONO predissociation. Alternatively, the whole HNO_2_ group might dissociate, forming the C_2_HN_3_^−^ anion with mass 67 u that was already discussed for TR^−^. This dissociation channel is more exothermic than the formation of [3NTR–NO_2_]^−^, but lower in intensity. This can be explained by the additional barrier that has to be overcome for opening the cycle in the C_2_HN_3_^−^ moiety (see Fig. 2). This ion might then further dissociate H or HCN to produce experimentally observed NCNCN^−^ or CN_2_^−^ anions (masses 66 u and 40 u), respectively. The ion with mass 41 u, interpreted as CHN_2_^−^, might be formed, *e.g.*, through the dissociation of the anion with the HONO moiety formed. All the discussed anions (masses 68 u, 67 u, 66 u, 41 u and 40 u) share the same features in the DEA experiment.

### Direct dissociation channels

Finally, the ions with masses 113 u and 16 u, assigned to [3NTR–H]^−^ and O^−^, may arise through direct dissociation. Direct N–H bond dissociation might take place already at low electron energies of ∼0.3 eV, in agreement with the experimental energies, forming the [3NTR–H]^−^ anion with the highest intensity. Formation of O^−^ is predicted to be possible only at energies higher than 2.3 eV. The experiment agrees with this prediction since anion formation starts at the threshold of 3.3 eV.

## Conclusions

We investigated the attachment of electrons to triazole and 3-nitrotriazole using mass spectrometry experiments and quantum chemistry. For triazole, five anionic fragments were recorded within dissociative electron attachment and the parent anion was not observed. The most prominent channel is simple N–H bond cleavage. A ring cleavage reaction plays a minor role which would support the view of triazoles as conjugative linkers for electron transfer processes. At high energies, a particularly stable dicyanamide anion, NCNCN^−^, is formed through subsequent dissociation of three hydrogen atoms. The DEA processes in 3-nitrotriazole are driven by the presence of the nitro group whose chemistry is involved in most dissociation channels. The most intense fragment corresponds again to the N–H dissociation channel. We suggest that predissociation of either NO_2_ or HNO_2_ moieties might take place upon electron attachment. These may then roam in the vicinity of the triazole cycle and interact with it to produce low-intensity fragments.

In terms of radiosensitization, the present results for the isolated molecule in the gas phase indicate that nitrotriazole indeed is generally an effective electron scavenger like the other nitro-group containing heteroaromatic molecules, nitrofuran and nitroimidazole. However, the decay of the transient negative ion formed is distinctly different for all three prototype systems. While for nitrofuran, the parent anion and NO_2_^−^ are the dominant anions,^39^ and for nitromidazole, the loss of neutral OH and NO upon DEA is most efficient,^38^ here preferentially the dehydrogenation, NO_2_^−^ and the loss of OH occur. Thus, the addition of NO_2_ is responsible for an increase of the attachment cross-section, but the outcome of the unimolecular dissociation processes remains a specific feature of each compound. Apart from the ion signals at an electron energy of about 0 eV, we also note a shift of the major resonance at ∼3–4 eV for nitrofuran and nitroimidazole to ∼1 eV for nitrotriazole. Future resonance scattering calculation may shed more light on this novel resonance which may also be not found in the native triazole molecule. Regarding radiosensitization of cells, a mechanism was suggested for nitroimidazoles which involves the formation of the intact parent radical anion, while the release of NO_2_^−^ would not be beneficial.^30,58^ Applying the current status of knowledge for electron attachment to nitroimidazoles in a molecular environment, the quenching of NO_2_^−^ would be probable for non-isolated 3NTR since energy-dissipating processes may interfere with the predissociation process. For the abundant direct emission of the hydrogen radical observed here, a prediction may not be straightforward (see for example ref. 59) and future experiments studying this reaction under more complex conditions are required.

## Author contributions

M. S. and F. I. carried out the measurements. L. U. I. and M. O. performed the quantum chemical calculations. S. D. conceived the study. M. O. and S. D. prepared the first manuscript draft.

## Conflicts of interest

There are no conflicts to declare.

## Supplementary Material

CP-025-D3CP01162C-s001
