# Supplementary material for: Decomposition of triazole and 3-nitrotriazole upon low-energy electron attachment
Source: Phys Chem Chem Phys. 2023 Apr 27;25(20):13892–901. doi: 10.1039/d3cp01162c (PMC10207873; doi:10.1039/d3cp01162c)
Supplement: CP-025-D3CP01162C-s001 [file CP-025-D3CP01162C-s001.pdf]

Electronic Supplementary Material (ESI) for Physical Chemistry Chemical Physics.
This journal is © the Owner Societies 2023

Supporting Information

Decomposition of triazole and 3-nitrotriazole upon low-energy electron attachment

Muhammad Saqib,[1,2] Farhad Izadi,[1,2] Leon U. Isierhienrhien,[1]

Milan Ončák,[1*] and Stephan Denifl[1,2*]

[1]Institut für Ionenphysik und Angewandte Physik, Universität Innsbruck, Technikerstraße 25, A-6020 Innsbruck, Austria

[2]Center for Biomolecular Sciences Innsbruck, Universität Innsbruck, Technikerstraße 25, A-6020 Innsbruck, Austria

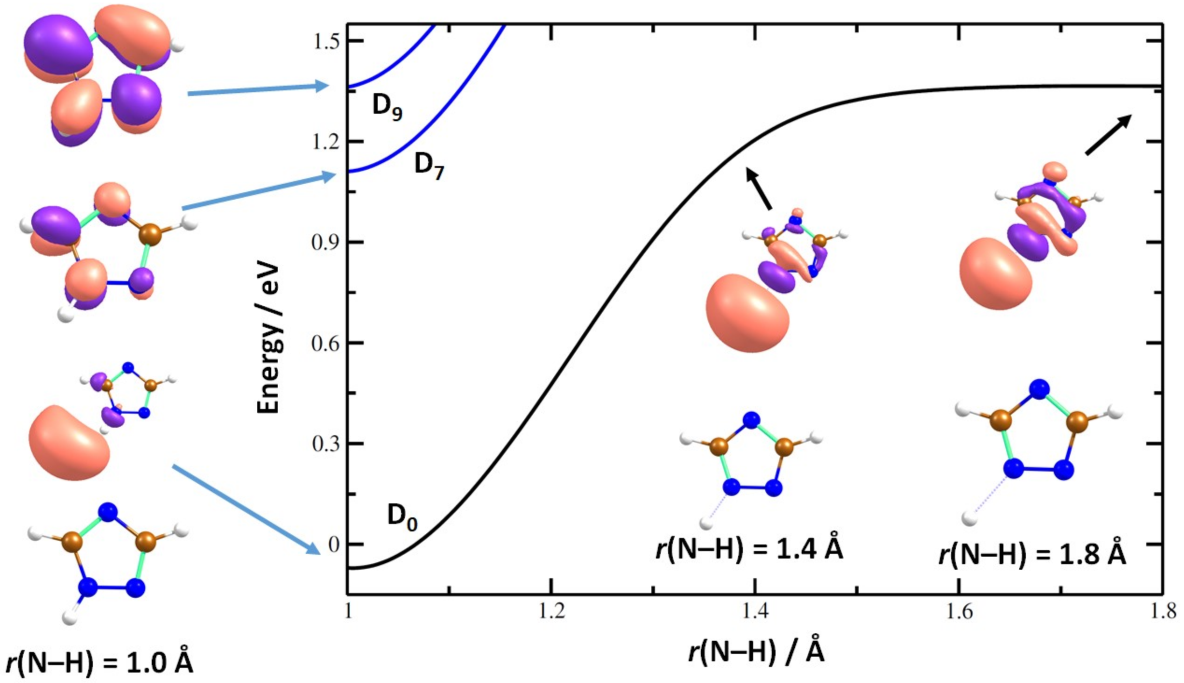

Figure S1 – Potential energy curves in TR⁻ along the N–H coordinate for the dipole-bound state of A' irreducible representation (black line) and states of the π* character of A'' irreducible representation (blue lines). Selected orbitals with the odd electron are shown as calculated within the natural transition orbital formalism. Calculated at the (TD-)CAM-B3LYP/aug-cc-pVTZ(H+)//B3LYP/aug-cc-pVTZ(H+) level of theory, the structures were optimized for the anion with the respective N–H distances kept constant. The energy is given with respect to the neutral TR molecule as calculated at the B3LYP/aug-cc-pVTZ(H+) level.

Cartesian coordinates (in Å) and electronic energies including zero-point correction (in Hartree) of structures optimized at the B3LYP/aug-cc-pVTZ level

```
3NTR-, TS for HNO2 formation
E = -446.896872
o 1.384670 -1.534524 -0.000000
h 0.057436 -1.660682 -0.000000
n 1.327250 -0.167723 -0.000000
c -0.000000 0.236699 0.000000
o 2.351976 0.562100 -0.000000
n -0.635346 1.408165 0.000000
c -1.923885 0.972993 0.000000
n -0.823718 -0.817728 0.000000
n -2.102961 -0.347000 0.000000
h -2.763875 1.651928 0.000000

3NTR-, HNO2 formed
E = -446.912306
o 1.888291 1.128897 -0.019819
h 1.084378 1.698037 -0.033127
n 1.300280 -0.165593 0.091680
c -0.095187 -0.126192 0.025067
o 2.055316 -1.142500 -0.059006
n -0.912631 -1.186534 0.018593
c -2.101420 -0.547011 -0.013951
n -0.698783 1.069129 0.004365
n -2.032794 0.786073 -0.024838
h -3.046108 -1.071519 -0.031567

C2N3H-
E = -241.058421
N -1.100667 0.283153 0.000000
C -0.000000 1.032145 -0.000000
N 1.171725 0.338626 -0.000000
C 0.665771 -0.892751 0.000000
N -0.635583 -1.043255 0.000000
H -0.042950 2.113978 -0.000000

C2N3H2-
E = -241.736112
N -0.714829 -0.942113 -0.000000
C 0.616786 -0.893037 -0.000000
N 1.135740 0.353970 0.000000
C 0.000000 1.085187 0.000000
N -1.123756 0.369170 0.000000
H -0.006901 2.167472 0.000000
H 1.226098 -1.787561 -0.000000

C2N3H2O-
E = -317.009444
C 0.775612 -1.234121 0.000000
N -0.522989 -1.472815 0.000000
N -1.014132 -0.181377 0.000000
C -0.000000 0.771433 0.000000
N 1.170495 0.056442 0.000000
O -0.195700 2.002656 0.000000
H -2.000632 -0.003722 0.000000
H 1.478938 -2.057145 0.000000

C2H2N4O
E = -371.618806
N -0.634914 -1.717849 0.000000
N 0.417864 -0.899084 -0.000000
C 0.000000 0.386931 0.000000
N -1.320899 0.432613 0.000000
C -1.664785 -0.870089 0.000000
N 0.839871 1.524393 -0.000000
O 2.024319 1.248396 -0.000000
H 1.363086 -1.247848 -0.000000
H -2.682382 -1.220887 0.000000

3NTR
E = -446.850826
N -2.184986 0.059113 0.000000
N -1.078027 -0.695384 0.000000
C -0.000000 0.104564 0.000000
N -0.340223 1.366555 0.000000
C -1.693665 1.292930 0.000000
N 1.342371 -0.447412 0.000000
O 1.401572 -1.674664 0.000000
O 2.277119 0.322033 0.000000
H -1.108465 -1.702599 0.000000
H -2.333020 2.158580 0.000000

3NTR-
E = -446.915585
N 2.092947 -0.665895 0.000000
N 0.768111 -0.998296 0.000000
C -0.000000 0.122844 0.000000
N 0.792111 1.204261 0.000000
C 2.027553 0.657113 0.000000
N -1.368560 0.024354 -0.000000
O -1.839605 -1.189514 -0.000000
O -2.098060 1.071134 -0.000000
H 0.417247 -1.938510 0.000000
H 2.926492 1.254840 0.000000

CH2CN-
E = -132.173972
C 0.043591 1.196113 -0.000000
C 0.000000 -0.183567 0.000000
N -0.001302 -1.362226 0.000000
H -0.126214 1.730153 0.925257
H -0.126214 1.730153 -0.925257

TR
E = -242.284062
C 1.075567 0.260632 -0.000000
N 0.687143 -0.997139 0.000000
C -0.669982 -0.896370 0.000000
N -1.137408 0.337607 0.000000
N -0.000000 1.069587 -0.000000
H -1.326020 -1.750080 0.000000
H 2.090800 0.620225 -0.000000
H -0.046440 2.073886 -0.000000

TR-
E = -242.275647
C 1.070726 0.264410 0.000000
N 0.686799 -1.001941 -0.000000
C -0.667977 -0.897473 -0.000000
N -1.136180 0.337468 -0.000000
N 0.000000 1.068060 0.000000
H -1.327205 -1.750823 -0.000000
H 2.081670 0.638841 0.000000
H -0.025289 2.085249 0.000000

C3CN-
E = -132.755277
N 0.000054 -1.433082 0.000000
C -0.000000 -0.282047 0.000000
```

C -0.000200 1.175793 -0.000000
H 1.024676 1.556208 -0.000000
H -0.511927 1.556444 0.887703
H -0.511927 1.556444 -0.887703

[3NTR-H]-
E = -446.338939
N -0.856880 2.010103 -0.000000
N -1.177079 0.704251 -0.000000
C 0.000000 0.068318 -0.000000
N 1.086521 0.861700 0.000000
C 0.492249 2.055767 -0.000000
N 0.088557 -1.364864 0.000000
O -0.955622 -2.020283 0.000000
O 1.208524 -1.881310 0.000000
H 1.035454 2.989902 -0.000000

HC2NO-
E = -206.774182
N -0.778449 0.830743 0.029389
C 0.249582 -0.030598 -0.021889
O 1.478781 -0.100469 0.022642
C -1.033384 -0.605082 -0.130195
H -1.678296 -1.197372 0.525643

C2N3-
E = -240.382837
N -0.642758 0.982996 0.000000
C 0.662620 0.818098 -0.000000
N 1.192958 -0.422552 -0.000000
C 0.000000 -1.052766 -0.000000
N -1.118160 -0.359300 0.000000

[3NTR-NO2]
E = -241.598295
N -1.088716 0.329019 0.000000
N -0.570364 -0.937978 0.000000
C 0.764098 -0.850461 -0.000000
N 1.177156 0.362176 -0.000000
C 0.000000 1.071147 -0.000000
H -1.170023 -1.743295 0.000000
H -0.041095 2.146663 -0.000000

C2N3H-, TS for ring opening
E = -241.037667
n -0.533056 -1.291998 0.000000
n -1.173473 0.502368 0.000000
c 0.000000 1.061155 -0.000000
c 0.623692 -0.895239 -0.000000
n 1.164039 0.340256 -0.000000
h 0.055273 2.150129 -0.000000

C2N3H-, open ring
E = -241.105866
n 2.096501 -0.458905 -0.000000
n -1.652438 -0.901029 0.000000
c -1.243732 0.307403 0.000000
c 1.066556 0.098566 -0.000000
n 0.000000 0.859853 -0.000000
h -2.045382 1.064757 0.000000

C2N3-, open ring
E = -240.574186
n -0.000000 2.234353 -0.383963
n -0.000000 -2.234353 -0.383963
c 0.000000 -1.150426 0.055962
c 0.000000 1.150426 0.055962
n -0.000000 -0.000000 0.671992

[3NTR-OH]-
E = -371.102133
N 1.518836 -1.214086 -0.000000
N 1.343961 0.102842 -0.000000
C 0.000000 0.299598 -0.000000
N -0.715768 -0.859835 0.000000
C 0.264191 -1.748071 -0.000000
N -0.460537 1.600740 0.000000
O -1.686992 1.762670 0.000000
H 0.105353 -2.818158 -0.000000

CH2O
E = -114.525654
C -0.000000 0.000000 -0.526976
O 0.000000 -0.000000 0.673248
H 0.000000 0.938294 -1.112065
H -0.000000 -0.938294 -1.112065

CH3
E = -39.829718
C -0.000000 0.000000 -0.000027
H -0.000000 1.077944 0.000054
H 0.933527 -0.538972 0.000054
H -0.933527 -0.538972 0.000054

NCNH-
E = -148.263535
N 0.019419 1.302846 0.000000
C 0.000000 0.119905 0.000000
N 0.097676 -1.175616 -0.000000
H -0.819666 -1.610041 -0.000000

CN2-
E = -147.629826
N 0.000000 0.000000 1.230244
C 0.000000 0.000000 -0.000010
N 0.000000 0.000000 -1.230235

NCNO2H
E = -298.575294
C -0.293001 -0.927705 0.000000
N -0.578461 -2.048383 0.000000
N -0.000000 0.377810 -0.000000
O -0.825862 1.310102 -0.000000
O 1.376891 0.645260 -0.000000
H 1.398990 1.617338 -0.000000

CN-
E = -92.890710
C 0.000000 0.000000 -0.630719
N 0.000000 0.000000 0.540617

CN
E = -92.742015
C 0.000000 0.000000 -0.625843
N 0.000000 0.000000 0.536437

NCH2
E = -93.996519
C 0.000000 -0.000000 -0.501357
N -0.000000 0.000000 0.736682
H 0.000000 0.935390 -1.074314
H -0.000000 -0.935390 -1.074314

CH2NH

E = -94.632234
C 0.056009 0.583348 0.000000
N 0.056009 -0.680566 -0.000000
H -0.842962 1.206064 0.000000
H 1.009051 1.110463 0.000000
H -0.894209 -1.052650 -0.000000

H2
E = -1.169964
H 0.000000 0.000000 0.371491
H 0.000000 0.000000 -0.371491

HCN
E = -93.446436
C 0.000000 0.000000 -0.496970
H 0.000000 0.000000 -1.562343
N 0.000000 0.000000 0.649166

H
E = -0.502260
H 0.000000 0.000000 0.000000

HNO2
E = -205.771706
N 0.000000 0.519276 0.000000
O -1.110597 0.173873 0.000000
O 0.890148 -0.604962 0.000000
H 1.763592 -0.186214 0.000000

HNO
E = -130.513712
H -0.941598 0.921268 0.000000
N 0.062773 0.577602 0.000000
O 0.062773 -0.620560 0.000000

NCNH2
E = -148.814628
N 0.082177 -1.116343 0.000000
C -0.000000 0.221202 -0.000000
N -0.015856 1.375738 -0.000000
H -0.232126 -1.571489 0.843859
H -0.232126 -1.571489 -0.843859

N2
E = -109.565010
N 0.000000 0.000000 0.545581
N 0.000000 0.000000 -0.545581

NCO-
E = -168.189174
C 0.000000 0.000000 -0.071402
N 0.000000 0.000000 -1.258406
O 0.000000 0.000000 1.154657

NO2
E = -205.102430
O -0.000000 0.971416 -0.240428
N -0.000000 0.000000 0.549549
O -0.000000 -0.971416 -0.240428

NO2-
E = -205.234332
N 0.000000 -0.000000 0.457244
O -0.000000 1.069664 -0.200044
O -0.000000 -1.069664 -0.200044

NO
E = -129.938759
O 0.000000 0.000000 0.534552
N 0.000000 0.000000 -0.610916

O-
E = -75.155815
O 0.000000 0.000000 0.000000

OH
E = -75.760185
O 0.000000 0.000000 0.108367
H 0.000000 0.000000 -0.866937

3NTR-, NO2 predissociation TS
E = -446.870681
c 0.280090 -0.372296 -0.171587
n -1.784281 -0.079826 0.230122
n 0.792996 0.866948 -0.119791
n 2.150583 0.853869 0.047259
c 2.379405 -0.447408 0.091674
n 1.279425 -1.242985 -0.027315
h 3.380021 -0.834297 0.216385
h 0.256603 1.717846 -0.133814
o -2.009630 1.124909 0.006547
o -2.573452 -0.968830 -0.070924

3NTR-, NO2 predissociation
E = -446.873421
c -0.602040 -0.699706 0.000011
n 2.004527 -0.151382 -0.000235
n -0.704684 0.630498 0.000012
n -2.013823 1.021881 0.000173
c -2.630159 -0.147777 0.000329
n -1.811498 -1.239818 0.000221
h -3.707352 -0.215582 0.000496
h 0.083412 1.270771 -0.000134
o 2.057452 1.095144 -0.000460
o 3.029483 -0.819962 0.000010

3NTR-, formation of C-O bond TS
E = -446.859337
o -1.269297 -0.959999 0.350876
c 0.217884 -0.343083 -0.370631
n 0.587146 0.918573 -0.050547
n 1.928572 0.992879 0.236155
c 2.284075 -0.269823 0.081131
n 1.293082 -1.131852 -0.266292
n -2.377390 -0.330532 0.067873
o -2.270093 0.885178 -0.150754
h -0.024238 1.712877 -0.003231
h 3.307735 -0.580368 0.228935

3NTR-, formation of C-O bond
E = -446.955197
o 0.724962 1.299763 0.533082
c -0.299787 0.641042 0.234832
n -0.538154 -0.655217 0.666261
n -1.727924 -1.148267 0.188258
c -2.164793 -0.107829 -0.500080
n -1.377915 0.987228 -0.527082
n 2.661307 0.162707 -0.274808
o 2.361781 -0.953845 -0.402324
h 0.088918 -1.237604 1.188900
h -3.116580 -0.154182 -1.011880

3NTR-, formation of C-H bond TS
E = -446.823580

```
h 0.475817 -0.758752 1.070444
c -0.044360 -0.139434 0.121817
n -0.903765 -1.019583 -0.560415
n -2.105046 -0.565971 -0.394145
c -1.981328 0.640955 0.283950
n -0.761224 0.969969 0.587527
h -2.854167 1.225346 0.538579
o 1.965484 -0.655206 0.892448
n 1.382225 -0.085428 -0.229115
o 1.940409 0.834127 -0.876272
```

3NTR-, formation of C-H bond
E = -446.899760

```
h 0.124887 -1.852859 -0.529256
c 0.724047 -0.993967 -0.270616
n 0.911700 -0.608062 1.014784
n 1.745210 0.445641 0.955416
c 2.012939 0.619505 -0.355599
n 1.408046 -0.258800 -1.168953
h 2.666405 1.405794 -0.706140
o -2.623793 -0.701991 0.045520
n -1.730212 0.110067 -0.057321
o -1.820759 1.310979 -0.072369
```

D0/D1 conical intersection in 3NTR-,
CASSCF(3,5)/aug-cc-pVDZ

```
N   0.00182538 -0.629663 -2.11584
N   0.00212239 -1.03602 -0.843201
C   0.000503958 0.0499416 -0.0966284
N   -0.00297838 1.1439 -0.741547
C   -0.00159343 0.673976 -2.03094
N   0.000296744 -0.000943501 1.40293
O   -0.00367036 -1.06306 1.87409
O   0.00339657 1.02179 1.95501
H   0.00310033 -2.01531 -0.605662
H   -0.00336653 1.30792 -2.89306
```
